# Coverage by examinations associated with early detection of colorectal neoplasia in the Czech Republic

**DOI:** 10.1093/eurpub/ckad071

**Published:** 2023-05-04

**Authors:** Ondřej Ngo, Kateřina Hejcmanová, Štěpán Suchánek, Lucie Pehalová, Ladislav Dušek, Miroslav Zavoral, Jan Bureš, Bohumil Seifert, Karel Hejduk, Norbert Král, Ondřej Májek

**Affiliations:** Institute of Health Information and Statistics of the Czech Republic, Prague, Czech Republic; Institute of Biostatistics and Analyses, Faculty of Medicine, Masaryk University, Brno, Czech Republic; Institute of Health Information and Statistics of the Czech Republic, Prague, Czech Republic; Institute of Biostatistics and Analyses, Faculty of Medicine, Masaryk University, Brno, Czech Republic; Military University Hospital, First Faculty of Medicine, Charles University, Prague, Czech Republic; Institute of Health Information and Statistics of the Czech Republic, Prague, Czech Republic; Institute of Biostatistics and Analyses, Faculty of Medicine, Masaryk University, Brno, Czech Republic; Institute of Health Information and Statistics of the Czech Republic, Prague, Czech Republic; Institute of Biostatistics and Analyses, Faculty of Medicine, Masaryk University, Brno, Czech Republic; Military University Hospital, First Faculty of Medicine, Charles University, Prague, Czech Republic; Military University Hospital, First Faculty of Medicine, Charles University, Prague, Czech Republic; Institute of General Practice, First Faculty of Medicine, Charles University, Prague, Czech Republic; Institute of Health Information and Statistics of the Czech Republic, Prague, Czech Republic; Institute of Biostatistics and Analyses, Faculty of Medicine, Masaryk University, Brno, Czech Republic; Institute of General Practice, First Faculty of Medicine, Charles University, Prague, Czech Republic; Institute of Health Information and Statistics of the Czech Republic, Prague, Czech Republic; Institute of Biostatistics and Analyses, Faculty of Medicine, Masaryk University, Brno, Czech Republic

## Abstract

**Background:**

Coverage by examinations is a crucial indicator of the future impact on the burden of colorectal cancer (CRC). The study aimed to evaluate coverage by examinations associated with CRC screening and early cancer detection of CRC in the Czech Republic. The burden of CRC was also assessed.

**Methods:**

The novel nationwide administrative registry with individual data (period 2010–19) was used to evaluate coverage by examinations for screening faecal occult blood test and colonoscopy. In the second step, additional examinations for early CRC detection were included in the coverage calculation (complete coverage). Age-specific trends in CRC incidence (period 1977–2018) were investigated using Joinpoint regression.

**Results:**

Coverage by screening examinations within recommended interval was around 30%. Complete coverage reached >37% and >50% at the 3-year interval. The coverage by examinations for the non-screening population aged 40–49 years was almost 4% and 5% (most of them were colonoscopies) at the 3-year interval. In age groups aged ≥50 years, we observed a significant annual decline, especially in the 50–69 age group, with recent annual decreases reaching up to 5–7%. The change in trend and the recent decline were also observed in the age group 40–49.

**Conclusions:**

More than half of the target screening population was covered by examinations potentially associated with early detection and subsequent treatment of colorectal neoplasms. The substantial coverage by potentially prophylactic examinations might be an explanation for the considerable decrease in CRC incidence.

## Introduction

Colorectal cancer (CRC) is among the most common cancers and cancer causes of death worldwide, with more than 1.8 million cases and 880 thousand deaths estimated for 2018.^1^ CRC screening with guaiac or immunochemical faecal occult blood test (FOBT) reduces CRC mortality^2^ and several studies have shown that screening based on FOBT can also reduce CRC incidence.^3^^,^^4^ Moreover, epidemiological studies have shown screening colonoscopy to decrease CRC incidence and mortality effectively.^5^^,^^6^ Usual screening strategies using immunochemical FOBT (annual or biennial) or colonoscopy (10 yearly) are cost-effective compared with no screening at reasonable willingness-to-pay thresholds.^7^

The participation rate is a key performance indicator that reflects the acceptability of a screening programme in the target population and is also directly related to the future impact of the programme on the population burden of CRC. Coverage by examinations is the method of choice for monitoring screening utilization in settings without universal invitation schemes. The estimate should be stratified by sex, age, screening modality and history.^8^

Removal of colorectal adenomas during colonoscopy examination for different indications in different settings was shown to be associated with decreased CRC incidence^9^ and mortality.^10^ We, therefore, did not limit our attention to FOBTs and colonoscopy examinations performed and reimbursed for screening purposes, but we also included examinations performed for other purposes (diagnosis, screening of high-risk individuals, surveillance, etc.), as these examinations are also potentially associated with opportunistic CRC screening and subsequent early neoplasia detection, potentially resulting in a decrease in CRC incidence and mortality.

Such complete coverage by examinations is usually tricky to estimate. Estimates of the volume of all colonoscopies performed were previously shown to be challenging to obtain and come mainly from surveys^11^ or ad hoc insurance cohorts.^12^ For this study, we used the recently established National Registry of Reimbursed Health Services (NRRHS), part of the National Health Information System of the Czech Republic.^13^ NRRHS aggregates data on reimbursed care from all public health insurance companies (covering all the population, screening according to national policy and associated diagnostic examinations are reimbursed and therefore included). Consequently, it allows us to cover all examinations provided within the National CRC screening programme and other examinations reimbursed within the public system.

Our study aimed to estimate coverage by examinations potentially associated with CRC screening or early cancer detection in the Czech Republic. At the same time, we aimed to estimate cumulative coverage by colorectal examinations during more prolonged periods. Indicators of CRC burden are also provided to show the national context. We also focused on younger age groups <50 years in the context of recent international studies that observed an increase in early-onset CRC incidence.^14^^,^^15^

## Methods

### Setting and study design

CRC screening programme was initiated in the Czech Republic in 2000 for individuals >50 years of age, with guaiac FOBT offered at a biennial interval. Since 2009, individuals aged 50–54 years were offered annual guaiac/immunochemical FOBT. Individuals >55 years were offered the option to choose screening colonoscopy in a 10-year interval instead of the available biennial guaiac/immunochemical FOBT.^16^ In case of a positive FOBT result, the person is referred for a colonoscopy. Since 2020, screening colonoscopy has been offered for individuals >50 years old. The upper age limit for screening has not yet been set in the Czech Republic and is usually evaluated by the registering physician with regard to the biological age, prognosis of the patient and potential individual benefit from screening. Individuals enter screening usually as a part of a regular check-up at their GP or primary care gynaecologist. Introducing personal invitation letters is an effective strategy for increasing CRC screening participation.^17^ To increase participation in the CRC screening programme in the Czech Republic, the personal invitation was introduced in the Czech Republic in 2014. Individuals invited include those <70 years old who had not undergone a recent colorectal examination.^18^ Thus, not all individuals in the target population are invited regularly, so a certain proportion of individuals do not attend screening programme at the recommended screening intervals.

The study used population-based individual data from national health registries to assess the coverage of the colorectal screening population (all persons aged ≥50 years) with screening examinations, as well as the coverage of other examinations related to early detection of CRC at different time intervals. At the same time, in response to an article assessing the increasing incidence in persons aged <50 years and the position of the Czech Republic, which is the only country with a significant decline in incidence in persons aged 40–49 years,^14^ coverage was similarly assessed in younger persons. Age-specific trends in CRC incidence were also evaluated in the context of a potential explanation for the decline in the population burden of CRC due to complete coverage by examinations.

### Data sources

Population-based statistics on coverage by examinations are based on individual data available from all health insurance companies in the Czech Republic (there are currently seven health insurance companies, all of which reimburse CRC screening and diagnosis). Data are collected in the NRRHS and contain national data from all healthcare providers (approximately 30 000) on individual payments of healthcare providers, data on health services providers, data on personnel, technical and material equipment of workplaces and the necessary metadata since 2010. Simply put, the registry contains all examinations reported and accepted for reimbursement purposes, medications and materials provided to a patient by a healthcare provider. For our analysis, we used individual data on reported screening and diagnostic examinations (FOBTs and colonoscopies) in 2010–19 for estimation coverage by examinations potentially associated with CRC screening or early cancer detection. The definition of the coverage calculation by examinations is described in more detail below.

The Czech National Cancer Registry (CNCR) is the primary data source on cancer epidemiology in the Czech Republic. CNCR has become an integral part of comprehensive cancer care and contains detailed records of all diagnosed cancers in the Czech population since 1977. Registration of tumours is stipulated by law and is obligatory.^19^ For our analysis, we used age-specific incidence rates of CRC up to 2018 from the CNCR and demographic data from the Czech Statistical Office. These epidemiological data are publicly available on the website www.svod.cz.^20^

### Statistical analysis

The primary outcome was coverage by examinations. In our study, we distinguished the examinations reported and reimbursed as CRC screening (coverage by screening examinations) and the coverage by examinations related in addition to early detection or diagnosis (coverage by examinations associated with CRC screening or early cancer detection or diagnosis, hereinafter referred to as complete coverage by examinations). The age of persons was determined for the year for which the coverage is assessed. The denominator represented the population at the end of the year for which coverage was calculated. If the persons were reported several different examinations during a defined interval, they were assigned only one of them, in the following order: screening colonoscopy (sC), screening FOBT (sFOBT; persons with a positive FOBT and follow-up colonoscopy were counted only once in this category), diagnostic colonoscopy (dC), diagnostic FOBT (dFOBT). For people <50 years of age, only dC or dFOBT are considered.

Coverage by screening examinations (sC or sFOBT) was calculated as the proportion of the number of persons aged ≥50 years with reported sC or sFOBT at the recommended screening interval: Proportion of persons who have undergone sC in the last 10 years or have undergone sFOBT in the last 2 years (in the age group 50–54 during the previous year).


$$\mathrm{Coverage}\;\mathrm{by}\;\mathrm{screening}\;\mathrm{examination}=\frac{\mathrm{persons}\;\mathrm{aged}\;50\;\mathrm{and}\;\mathrm{over}\;\mathrm{with}\;\mathrm{reported}\;\mathrm{sC}\;\mathrm{or}\;\mathrm{sFOBT}\;}{\mathrm{persons}\;\mathrm{aged}\;50\;\mathrm{and}\;\mathrm{over}\;\mathrm{in}\;\mathrm{the}\;\mathrm{population}\;}$$

Complete coverage by examinations was defined as the proportion of the number of persons aged ≥50 years who were examined by sC, or sFOBT, or dC, or dFOBT.

Complete coverage by examinations at the recommended interval was calculated as the proportion of persons who have undergone screening colonoscopy in the last 10 years, or have undergone sFOBT, or dC, or dFOBT during the last 2 years. For persons aged 50–54 years and only in the case of sFOBT and dFOBT, a 1-year interval is applied.


$$\mathrm{Complete}\;\mathrm{coverage}=\frac{\mathrm{persons}\;\mathrm{aged}\;50\;\mathrm{and}\;\mathrm{over}\;\mathrm{examined}\;\mathrm{by}\;\mathrm{sC},\;\mathrm{sFOBT},\;\mathrm{dC}\;\mathrm{or}\;\mathrm{dFOBT}\;}{\mathrm{persons}\;\mathrm{aged}\;50\;\mathrm{and}\;\mathrm{over}\;\mathrm{in}\;\mathrm{the}\;\mathrm{population}\;}$$

Complete coverage by examinations was further assessed at 3-year, 4-year and 5-year intervals: Proportion of persons who have undergone sC in the last 10 years (for sC, the definition is the same for each interval), or have undergone sFOBT, or dC, or dFOBT during the last3, 4 or 5 years, respectively (for simplicity, the same interval was considered for sFOTB, dC, dFOBT and all ages—e.g. for the 3-year interval, it was considered whether individuals had undergone sFOTB or dC or dFOBT in the last 3 years if they had not undergone sC). For persons <50 years of age was considered a dS or dFOBT performed in 2-, 3-, 4- and 5-year intervals. For the coverage in the 2-year/recommended interval, the first calculation for 2011 was presented due to the fact that the last 2 years 2010 and 2011 are included in the calculation (all data including absolute numbers in the result tables for each year already represent the sum of examinations for that interval). For the 3-, 4- and 5-year intervals, the first calculation was presented in 2012, 2013 and 2014, respectively. All statistical analysis of coverage by examinations was performed using IBM SPSS 25 statistic software.

Age-specific epidemiological trends in CRC incidence within the period 1977–2018 were investigated using Joinpoint regression analyses.^21^ Joinpoint analysis uses an algorithm to define segments where statistically significant changes in temporal trends occur on a logarithmic scale (*P*-values <0.05 was considered statistically significant). The annual percentage change (APC) in each Joinpoint segment represents the rate of change in cancer incidence per year in each time period and specific age subgroup. The average annual percentage change (AAPC) for each age group was also evaluated for the period after the introduction of screening (2000–18), for the period over the last 10 (2009–18) and 5 (2014–18) years associated with the change in programme design and the introduction of personalized invitation. Joinpoint regression analyses were performed using Joinpoint Regression Program, National Cancer Institute.

## Results

The coverage by screening examinations of the target population (>50 years old) at recommended screening interval was 30.1% (31.7% in women and 28.2% in men) in 2019. Most people had undergone sFOBT and the contribution of sC to coverage was approximately 2 percentage points (pp). The highest coverage was observed in the age group 65–69 years (39.6% for women and 35.1% for men), while the lowest coverage (<20%) was observed in the youngest and the oldest age groups.

If we consider other examinations associated with CRC early detection (dFOBT or dC) together with screening examinations for the same interval (table 1), then the complete coverage by examinations in 2019 reached 37.2% (38.1% in women and 36.1% in men) for the persons aged ≥50 years. The highest contribution, in addition to the screening examinations, to the complete coverage was made by diagnostic colonoscopies (5.5 pp, contribution of dC was higher in men with 6.3 pp compared to women with 4.8 pp), followed by diagnostic FOBTs (1.6 pp, same value for both sexes). The coverage by examinations related to early detection for persons aged 40–49 years was 3.7% (3.1 pp were dC).

**Table 1:** Number of persons examined by screening examinations (sFOBT or sC) or diagnostic examinations (dFOBT or dC) during a recommended screening interval with a calculation of coverage/complete coverage by examinations for 2019 according to age and gender

Age group (years)		sC (N)	sC (%^a^)	sFOBT (N)	sFOBT (%^a^)	Coverage (%)	dC (N)	dC (%^a^)	dFOBT (N)	dFOBT (%^a^)	Complete coverage (%)
**Men**	30–34	–	–	–	–	**–**	6356	1.7	497	0.1	**1.8**
	35–49	–	–	–	–	**–**	8203	2.1	739	0.2	**2.3**
	40–44	–	–	–	–	**–**	13 312	2.8	3051	0.6	**3.4**
	45–49	–	–	–	–	**–**	14 695	3.4	3057	0.7	**4.1**
	50–54	1315	0.4	50 433	14.4	**14.8**	18 349	5.1	3363	1.0	**21.0**
	55–59	5927	1.8	92 780	28.3	**30.1**	15 825	4.8	5280	1.6	**36.5**
	60–64	10 435	3.3	91 325	28.9	**32.2**	18 581	5.9	5340	1.7	**39.7**
	65–69	11 304	3.6	99 577	31.5	**35.1**	22 377	7.1	5927	1.9	**44.1**
	70–74	8114	3.1	79 786	30.0	**33.1**	21 380	8.1	4720	1.8	**42.9**
	75–79	3760	2.2	46 071	27.5	**29.7**	14 359	8.6	2889	1.7	**40.0**
	80–84	1324	1.5	20 748	24.0	**25.6**	6137	7.1	1476	1.7	**34.4**
	85+	460	0.7	10 722	17.1	**17.8**	2779	4.4	1004	1.6	**23.8**
	**Total 50+**	42 639	2.3	491 442	26.0	**28.2**	119 787	6.3	29 999	1.6	**36.1**
**Women**	30–34	–	–	–	–	**–**	5784	1.7	567	0.2	**1.8**
	35–49	–	–	–	–	**–**	7233	2.0	868	0.2	**2.2**
	40–44	–	–	–	–	**–**	12 262	2.7	2077	0.5	**3.2**
	45–49	–	–	–	–	**–**	14 097	3.5	2590	0.6	**4.1**
	50–54	875	0.3	62 561	18.6	**18.9**	15 352	4.6	3462	1.0	**24.5**
	55–59	4898	1.5	113 068	34.7	**36.2**	13 090	4.0	5452	1.7	**41.9**
	60–64	9767	2.9	117 391	35.0	**37.9**	15 661	4.7	6003	1.8	**44.4**
	65–69	10 942	3.0	132 873	36.6	**39.6**	19 528	5.4	6825	1.9	**46.8**
	70–74	8425	2.5	113 701	33.7	**36.2**	19 873	5.9	5837	1.7	**43.8**
	75–79	4129	1.7	70 977	29.4	**31.2**	14 191	5.9	4129	1.7	**38.8**
	80–84	1394	0.9	34 226	23.1	**24.0**	6669	4.5	2361	1.6	**30.1**
	85+	478	0.3	20 854	14.5	**14.8**	3451	2.4	1910	1.3	**18.6**
	**Total 50+**	40 908	1.8	665 651	29.8	**31.7**	107 815	4.8	35 979	1.6	**38.1**
**Total 50+**		86 547	2.0	1 157 093	28.1	**30.1**	227 602	5.5	65 978	1.6	**37.2**

sFOBT: screening FOBT; sC: screening colonoscopy; dFOBT: diagnostic FOBT; dC: diagnostic colonoscopy.

For people aged 50–54 years, a 1-year FOBT interval is considered.

For persons <50 years of age was considered dS or dFOBT performed in 2-year interval.

Values in bold represent coverage (sC, or sFOBT) and complete coverage (sC, or sFOBT, or dC, or dFOBT) for each age group and total for 50+.

a Contribution to complete coverage by examinations (percentage points).

If we consider the complete coverage by examinations within a longer 3-year interval (table 2, more detailed data are presented in Supplementary table S2), then it reached 50.0% (51.2% in women and 48.6% in men) in 2019 for persons aged ≥50 years. The most considerable contribution to the complete coverage within the extended 3-year interval was by sFOBT (39.2 pp, 41.4 pp in women and 36.6 pp in men), followed by dC (6.8 pp, 6.0 pp in women and 7.8 pp in men), dFOBT (2.0 pp, same value for both sexes) and screening colonoscopies (2.0 pp, 1.8 pp in women and 2.3 pp in men). The coverage by examinations related to early detection for persons aged 40–49 years was 5% (4.3 pp were dC).

**Table 2:** Coverage by individual examinations for age categories 30–39 years, 40–49 years and ≥50 years according to different time intervals over time

			Years								
Time interval for calculation coverage by examinations	Age (years)	Type of examination	2011	2012	2013	2014	2015	2016	2017	2018	2019
2-year interval	**30–39**	dC (%)	1.7	1.7	1.8	1.8	1.8	1.8	1.8	1.8	1.9
		dFOBT (%)	0.2	0.3	0.2	0.2	0.2	0.2	0.2	0.2	0.2
		**Total (%)**	**2.0**	**2.0**	**2.0**	**2.0**	**2.0**	**2.0**	**2.0**	**2.0**	**2.0**
	**40–49**	dC (%)	2.8	2.7	2.8	2.8	2.9	3.0	3.0	3.0	3.1
		dFOBT (%)	0.6	0.6	0.6	0.6	0.6	0.6	0.6	0.6	0.6
		**Total (%)**	**3.4**	**3.3**	**3.4**	**3.5**	**3.5**	**3.6**	**3.6**	**3.6**	**3.7**
Recommended interval	**50+**	sC (%)	0.2	0.3	0.4	0.7	1.0	1.2	1.5	1.8	2.0
		sFOBT (%)	24.5	25.2	26.1	30.4	31.7	29.6	28.8	28.4	28.1
		dC (%)	5.0	5.0	5.1	5.4	5.6	5.7	5.7	5.5	5.5
		dFOBT (%)	1.7	1.7	1.6	2.0	2.0	1.8	1.6	1.6	1.6
		**Total (%)**	**31.4**	**32.1**	**33.2**	**38.4**	**40.3**	**38.3**	**37.6**	**37.3**	**37.2**
3-year interval	**30–39**	dC (%)		2.5	2.5	2.5	2.5	2.5	2.6	2.6	2.6
		dFOBT (%)		0.3	0.3	0.3	0.3	0.3	0.3	0.2	0.2
		**Total (%)**		**2.8**	**2.8**	**2.8**	**2.8**	**2.8**	**2.8**	**2.8**	**2.8**
	**40–49**	dC (%)		3.9	3.9	3.9	4.0	4.1	4.2	4.2	4.3
		dFOBT (%)		0.8	0.8	0.8	0.8	0.8	0.8	0.8	0.8
		**Total (%)**		**4.6**	**4.6**	**4.7**	**4.8**	**4.9**	**4.9**	**5.0**	**5.0**
	**50+**	sC (%)		0.3	0.4	0.7	1.0	1.2	1.5	1.8	2.0
		sFOBT (%)		34.5	35.3	39.6	41.9	42.7	40.5	39.8	39.2
		dC (%)		6.0	6.1	6.3	6.5	6.7	6.8	6.8	6.8
		dFOBT (%)		2.3	2.1	2.4	2.5	2.4	2.2	2.1	2.0
		**Total (%)**		**43.0**	**43.9**	**49.0**	**51.9**	**53.1**	**51.0**	**50.5**	**50.0**
4-year interval	**30–39**	dC (%)			3.1	3.1	3.2	3.2	3.2	3.2	3.2
		dFOBT (%)			0.4	0.4	0.4	0.4	0.3	0.3	0.3
		**Total (%)**			**3.5**	**3.5**	**3.5**	**3.5**	**3.5**	**3.5**	**3.5**
	**40–49**	dC (%)			4.9	4.9	5.0	5.1	5.2	5.2	5.3
		dFOBT (%)			0.9	0.9	0.9	0.9	0.9	0.9	0.9
		**Total (%)**			**5.8**	**5.8**	**5.9**	**6.0**	**6.1**	**6.1**	**6.2**
	**50+**	sC (%)			0.4	0.7	1.0	1.2	1.5	1.8	2.0
		sFOBT (%)			40.5	44.5	46.7	47.8	48.0	46.3	45.5
		dC (%)			6.8	7.0	7.2	7.3	7.4	7.6	7.7
		dFOBT (%)			2.5	2.6	2.7	2.6	2.5	2.4	2.2
		**Total (%)**			**50.1**	**54.7**	**57.5**	**58.9**	**59.5**	**58.1**	**57.5**
5-year interval	**30–39**	dC (%)				3.7	3.7	3.7	3.8	3.8	3.8
		dFOBT (%)				0.5	0.5	0.4	0.4	0.4	0.4
		**Total (%)**				**4.2**	**4.2**	**4.2**	**4.2**	**4.1**	**4.1**
	**40–49**	dC (%)				5.7	5.8	6.0	6.1	6.1	6.3
		dFOBT (%)				1.1	1.1	1.0	1.0	1.0	1.0
		**Total (%)**				**6.8**	**6.9**	**7.0**	**7.1**	**7.1**	**7.2**
	**50+**	sC (%)				0.7	1.0	1.2	1.5	1.8	2.0
		sFOBT (%)				48.0	50.1	51.3	51.7	51.8	50.4
		dC (%)				7.4	7.5	7.7	7.8	8.0	8.2
		dFOBT (%)				2.7	2.8	2.7	2.6	2.5	2.4
		**Total (%)**				**58.8**	**61.4**	**62.9**	**63.6**	**64.1**	**63.0**

The values in bold represent the complete coverage for a given time interval, age group and year.

Table 2 shows the coverage of individual examinations according to different time intervals (recommended, 3, 4 and 5 years) in time trends separately for persons aged 30–39 years, 40–49 years and ≥50 years. For persons aged ≥50 years, the complete coverage in the recommended, 3-, 4- and 5-year periods in recent years was 37.2% (5.5 pp contribution of diagnostic colonoscopies), 50.0% (6.8 pp dC), 57.5% (7.7 pp dC) and 63.0% (8.2 pp dC), respectively. For people aged 40–49 years, the coverage by diagnostic procedures was ∼4% (3.1 pp dC), 5% (4.3 pp dC), 6% (5.3 pp dC) and >7% (6.3 pp dC), respectively.

**Figure 1:**
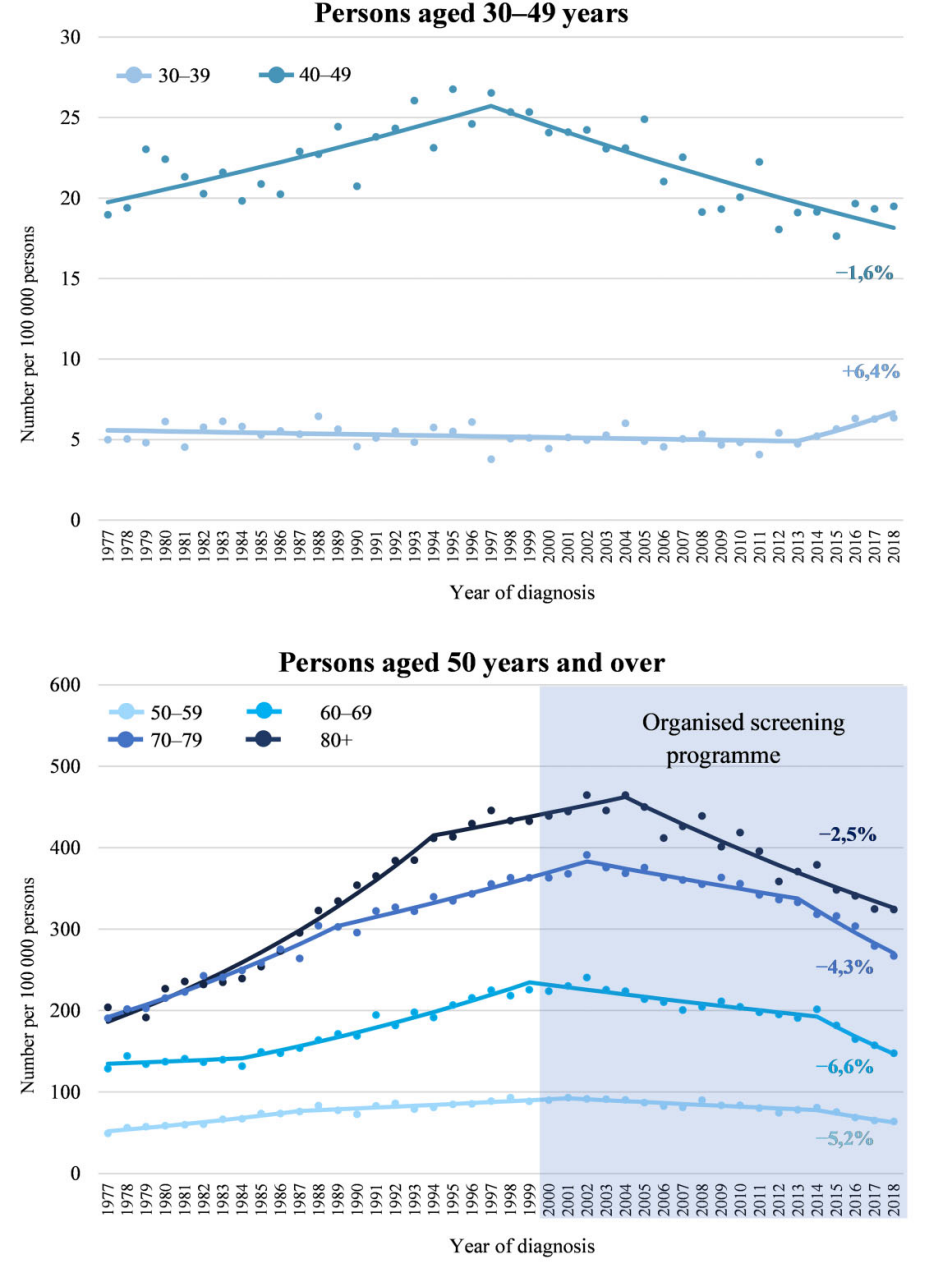
Joinpoint regression of CRC incidence in persons aged 30–49 years and ≥50 years—visualization of regression segments. Percentage changes describe the AAPC in the recent period 2014–18

In the past decade, we have observed a substantial decrease in CRC incidence in the Czech Republic throughout the age groups >40 years. Whereas before 2000, we generally saw an increase in incidence, the incidence started to decrease after 2000, with the incidence decreasing more quickly in the latest years (figure 1).

Shortly after 2000, we observed annual decreases of around 1–2% in most age groups. Recent annual declines reached up to 5–7% in age groups 50–69 in recent years (table 3). The change in trend and the recent decrease was also observed in the age group 40–49 but compared with most age groups ≥50 years, the annual percentage change for this age group has been stable over time since 2000, with an annual decline of 1.6%.

**Table 3:** Joinpoint regression of CRC incidence by age—APC in segments, AAPC for periods after screening implementation

Age group (years)	Segment	**APC (95% CI)**	AAPC 2000–18 (95% CI)	AAPC 2009–18 (95% CI)	AAPC 2014–18 (95% CI)
30–39	1977–2013	−0.4 (−0.7, −0.0)	+1.5 (−0.2, +3.2)	+3.3 (−0.1, +6.9)	+6.4 (−0.1, +13.2)
	2013–18	+6.4 (−0.1, +13.2)			
40–49	1977–97	+1.3 (+0.8, +1.8)	−1.6 (−2.1, −1.2)	−1.6 (−2.1, −1.2)	−1.6 (−2.1, −1.2)
	1997–2018	−1.6 (−2.1, −1.2)			
50–59	1977–87	+4.0 (+2.9, +5.1)	−2.0 (−2.9, −1.2)	−3.1 (−4.6, −1.5)	−5.2 (−8.6, −1.6)
	1987–2001	+1.3 (+0.8, +1.9)			
	2001–14	−1.3 (−1.9, −0.7)			
	2014–18	−5.2 (−8.6, −1.6)			
60–69	1977–84	0.7 (−0.8, +2.2)	−2.5 (−3.1, −1.9)	−3.7 (−4.8, −2.6)	−6.6 (−9.0, −4.1)
	1984–99	+3.4 (+3.0, +3.9)			
	1999–2014	−1.3 (−1.7, −0.9)			
	2014–18	−6.6 (−9.0, −4.1)			
70–79	1977–89	+3.9 (+3.4, +4.4)	−1.7 (−2.2, −1.3)	−2.9 (−3.6, −2.2)	−4.3 (−5.6, −3.0)
	1989–2002	+1.8 (+1.4, +2.2)			
	2002–13	−1.1 (−1.6, −0.7)			
	2013–18	−4.3 (−5.6, −3.0)			
80+	1977–94	+4.8 (+4.3, +5.3)	−1.7 (−2.0, −1.3)	−2.5 (−2.9, −2.1)	−2.5 (−2.9, −2.1)
	1994–2004	+1.1 (+0.2, +2.0)			
	2004–18	−2.5 (−2.9, −2.1)			

APC: annual percent change; AAPC: average annual percent change; CI: confidence interval.

## Discussion

Suboptimal colonoscopy quality with lower adenoma detection rates is associated with higher interval cancer rates.^22^ Whereas examinations occurring outside organized screening programme may be associated with lower quality, effectiveness and cost-effectiveness, these examinations are likely to still offer an important opportunity to visualize colon mucosa and potentially perform endoscopic polypectomy. These examinations could therefore offer a potential explanation for decreasing CRC incidence observed in the Czech population in recent years and possibly also the impact on incidence trends in people >50 years. Screening colonoscopies and follow-up colonoscopies after a positive FOBT, with an FOBT positivity rate of ∼6–7%,^23^ also have an impact on the population burden of CRC in the screening target population.

We can compare our results with the results of CRC screening in other countries. In the USA, the complete coverage by stool‐based tests at 1-year, flexible sigmoidoscopy at 5-year, or colonoscopy at 10-year intervals reached 62.6% in 2015.^24^ This corresponds to complete coverage at 5-year intervals in the Czech Republic.

Individual invitation of non-attenders, along with a one-time intensive mass-media campaign was introduced in the Czech Republic in 2014. This was likely associated with a substantial increase in coverage by examinations. Unfortunately, the effect has been fading out in recent years. The highest coverage was reached at the recommended interval in 2015, the 3-year interval in 2016, the 4-year interval in 2017 and the 5-year interval in 2018.

The results of the complete coverage by examinations presented above could be a potential explanation, along with innovations in the diagnosis and treatment of CRC, of the favourable trends in CRC burden. In 2007–17, the incidence decreased by 21.9% and mortality by 29.0%. In the same period, the coverage by FOBT at recommended screening interval increased from ∼16% to almost 29%. In addition, in 2015–17, one of the screening or diagnostic examinations was performed on more than half of the persons (in 2010–12 it was only 43.1%).From the historical data on colorectal screening coverage based on aggregated data, an increase in coverage of the target population by screening examinations (screening colonoscopy and screening FOBT) to almost 23%^16^ was observed in 2010 (the programme met all the criteria of an organized programme in 2009). If we consider a similar proportion of dC and dFOBT according to the results of the present study, the extrapolated complete coverage would be >35%, which could have an observable impact on incidence after about 5 years,^25^ which could be observed from 2013 to 2014 onwards in the form of a more substantial reduction in incidence.

Increasing coverage, possibly along with the improved quality of care, could likely be one factor contributing to the reduction of the epidemiological burden in the Czech Republic. This is in line with the results of the recent international analysis,^26^ where decreases in CRC burden were associated with early adoption of CRC screening practice. Interestingly, contrary to another international study,^14^ CRC incidence was also decreasing in younger adults aged 40–49 years, which may be again associated with the availability of prophylactic colonoscopy as a ‘grey’ screening modality in the Czech Republic.

This analysis was possible thanks to the availability of the NRRHS within the National Health Information System of the Czech Republic. This is a novel administrative data source allowing for long-term monitoring of the health system in the Czech Republic allowing for data linkages between individual registries, which presents a beneficial but still underutilized option for monitoring and evaluation of cancer screening.^27^ The key strength of this study is the long-term individual monitoring of examination histories, which provides a new way of assessing coverage by examinations and potentially explains favourable trends in CRC burden not only through the effect of regular screening examinations.

We realize that a possible limitation of the study might be the quality of the data, which depends on the reports of healthcare providers to health insurance companies. Some examinations might be reported incorrectly, namely with respect to classification between screening and diagnostic examinations. However, the health insurance data collection presents an official title for reimbursement and is subject to controlling and revision operations by the health insurance companies. Therefore, we assume that the impact of inaccuracies on the conclusions of this analysis regarding complete coverage (which considers examinations not distinguishing between the indications reported) is low.

It should also be taken into account that the diagnostic procedures included in our study may be indicated for diverse clinical reasons and may not all lead to early detection of cancer. Although diagnostic examinations performed because of symptoms of late-stage CRC did not lead to early detection, they constitute only a small number of included examinations (3000–4000 late CRCs are detected annually).^20^ On the other hand, substantial part of these examinations are performed due to unspecific symptoms not caused by late CRC and these can actually contribute to earlier detection and treatment of colorectal neoplasia. These examinations and other examinations (indicated because of surveillance reasons, screening of high-risk individuals and grey opportunistic screening) may play a potential role in explaining, for example, the decline in incidence in people <50 years of age. The observation of substantial coverage by examinations and concurrent decrease in CRC burden is an interesting finding. However, such association may be subject to bias and this hypothesis needs to be confirmed by further research.

In conclusion, our study showed that the coverage by all examinations potentially associated with early detection and treatment of colorectal neoplasms reached substantially higher values than the coverage only by screening FOBT or colonoscopy. In 2019, the complete coverage reached 37.2% at the recommended interval and even 50.0% at the 3-year interval. The substantial coverage of the target population by potentially prophylactic examinations might have been an explanation for the considerable concurrent decrease in CRC incidence.

## Supplementary Material

ckad071_Supplementary_Data

## Data Availability

The study is based on the data of National Health Information System and the authors are, according to current Czech laws, not authorized to share any potentially identifiable patient-level data. In justified cases, data may be formally requested through the corresponding author and the request for data will be assessed by the Institute of Health Information and Statistics of the Czech Republic. National linkable individual data are crucial for detailed evaluation of screening programmes through tracking of individual trajectories in the health system. The present study used a novel approach to assess population-based coverage by examinations related to CRC screening and early detection. The complete coverage by examinations (including screening and diagnostic colonoscopies and FOBT) of the target colorectal screening population indicates a much higher population coverage and could be a possible explanation for the declining incidence of the disease.
