# Supplementary material for: Coverage by examinations associated with early detection of colorectal neoplasia in the Czech Republic
Source: Eur J Public Health. 2023 May 4;33(3):515–21. doi: 10.1093/eurpub/ckad071 (PMC10234646; doi:10.1093/eurpub/ckad071)
Supplement: ckad071_Supplementary_Data [file ckad071_supplementary_data.docx]

# SUPPLEMENTARY RESULTS

Supplementary Table 1 shows the number of people examined by sFOBT or sC (which increased to more than 1.2 million following the implementation of personalised invitation of the target population for screening in 2014) over time and a demonstration of the calculation of coverage by screening examinations in the last year, which was described in the Results of the manuscript.

**Supplementary Table 1: Number of persons examined by sFOBT or sC during the recommended screening interval over time with a calculation of coverage by screening examinations for 2019 according to age and gender**

|  | | **Year** | | | | | | | | |  | | |  | |
| --- | --- | --- | --- | --- | --- | --- | --- | --- | --- | --- | --- | --- | --- | --- | --- |
| **Age group** | | **2011** | **2012** | **2013** | **2014** | **2015** | **2016** | **2017** | **2018** | **2019** | | | | | |
|  |  |  |  |  |  |  |  |  |  | **Number of sFOBT** | | **Number of sC** | **Target population** | | **Coverage** |
| **Men** | **50-54** | 36,739 | 37,355 | 39,446 | 57,339 | 55,417 | 54,914 | 53,890 | 53,420 | 50,433 | | 1,315 | 349,879 | | **14.8%** |
|  | **55-59** | 87,533 | 88,547 | 89,843 | 104,074 | 109,953 | 100,023 | 94,827 | 95,677 | 92,780 | | 5,927 | 328,076 | | **30.1%** |
|  | **60-64** | 91,641 | 92,938 | 94,163 | 113,312 | 123,014 | 113,501 | 108,719 | 106,217 | 91,325 | | 10,435 | 316,420 | | **32.2%** |
|  | **65-69** | 74,228 | 81,458 | 87,533 | 110,515 | 124,529 | 112,756 | 109,974 | 110,117 | 99,577 | | 11,304 | 315,679 | | **35.1%** |
|  | **70-74** | 46,978 | 50,784 | 56,650 | 68,937 | 76,246 | 77,344 | 81,610 | 85,104 | 79,786 | | 8,114 | 265,523 | | **33.1%** |
|  | **75-79** | 29,083 | 29,906 | 31,631 | 35,512 | 40,092 | 41,707 | 43,905 | 46,742 | 46,071 | | 3,760 | 167,590 | | **29.7%** |
|  | **80-84** | 16,185 | 17,221 | 18,400 | 20,374 | 21,526 | 21,019 | 20,647 | 21,289 | 20,748 | | 1,324 | 86,332 | | **25.6%** |
|  | **85+** | 6,356 | 6,939 | 7,767 | 9,140 | 10,172 | 10,427 | 10,666 | 10,885 | 10,722 | | 460 | 62,853 | | **17.8%** |
|  | **Total** | 388,743 | 405,148 | 425,433 | 519,203 | 560,949 | 531,691 | 524,238 | 529,451 | 491,442 | | 42,639 | 1,892,352 | | **28.2%** |
| **Women** | **50-54** | 52,728 | 53,588 | 55,999 | 71,488 | 68,173 | 67,270 | 65,732 | 64,247 | 62,561 | | 875 | 336,357 | | **18.9%** |
|  | **55-59** | 120,666 | 120,093 | 121,453 | 132,942 | 133,344 | 120,972 | 115,521 | 115,032 | 113,068 | | 4,898 | 325,425 | | **36.2%** |
|  | **60-64** | 128,507 | 129,579 | 132,908 | 152,389 | 158,302 | 145,391 | 140,396 | 135,158 | 117,391 | | 9,767 | 335,147 | | **37.9%** |
|  | **65-69** | 104,259 | 113,881 | 122,322 | 147,313 | 160,631 | 148,979 | 145,597 | 144,794 | 132,873 | | 10,942 | 363,248 | | **39.6%** |
|  | **70-74** | 67,366 | 73,109 | 81,513 | 96,833 | 106,344 | 109,362 | 115,825 | 116,441 | 113,701 | | 8,425 | 337,451 | | **36.2%** |
|  | **75-79** | 45,273 | 46,734 | 49,225 | 55,098 | 60,719 | 63,456 | 66,485 | 70,612 | 70,977 | | 4,129 | 241,039 | | **31.2%** |
|  | **80-84** | 27,374 | 29,312 | 30,767 | 33,806 | 35,411 | 34,403 | 34,132 | 34,472 | 34,226 | | 1,394 | 148,163 | | **24.0%** |
|  | **85+** | 13,478 | 14,892 | 15,949 | 17,930 | 19,791 | 20,388 | 20,417 | 20,857 | 20,854 | | 478 | 143,752 | | **14.8%** |
|  | **Total** | 559,648 | 581,188 | 610,136 | 707,799 | 742,715 | 710,221 | 704,105 | 704,613 | 665,651 | | 40,908 | 2,230,582 | | **31.7%** |
| **TOTAL** | | **948,391** | **986,336** | **1,035,569** | **1,227,002** | **1,303,664** | **1,241,912** | **1,228,343** | **1,234,064** | **1,157,093** | | **83,547** | **4,122,934** | | **30.1%** |

Supplementary Table 2 reflects the number of people examined by individual examinations related to CRC screening (sFOBT or sC) or early cancer detection (dFOBT or dC) during the three-year interval and their contribution to complete coverage by examinations for 2019 by age and gender.

**Supplementary Table 2: Number of persons examined by screening examinations (sFOBT or sC) or diagnostic examinations (dFOBT or dC) during the three-year interval with a calculation of coverage/complete coverage by examinations for 2019 according to age and gender**

|  |  |  |  | |  | |  | | | | | | | |
| --- | --- | --- | --- | --- | --- | --- | --- | --- | --- | --- | --- | --- | --- | --- |
| **Age group** | | **sC (N)** | | **sC (%^a^)** | | **sFOBT (N)** | | **sFOBT (%^a^)** | **Coverage** | **dC (N)** | **dC (%^a^)** | **dFOBT (N)** | **dFOBT (%^a^)** | **Complete coverage** |
| **Men** | **30-34** | - | | - | | - | | - | **-** | 8,753 | 2.4 % | 666 | 0.2 % | **2,5 %** |
|  | **35-49** | - | | - | | - | | - | **-** | 11,262 | 2.9 % | 967 | 0.2 % | **3,1 %** |
|  | **40-44** | - | | - | | - | | - | **-** | 18,326 | 3.9 % | 3,496 | 0.7 % | **4,6 %** |
|  | **45-49** | - | | - | | - | | - | **-** | 20,314 | 4.7 % | 3,682 | 0.9 % | **5,6 %** |
|  | **50-54** | 1,315 | | 0.4 % | | 106,395 | | 30.4 % | **30.8%** | 19,226 | 5.5 % | 6,638 | 1.9 % | **38,2 %** |
|  | **55-59** | 5,927 | | 1.8 % | | 123,780 | | 37.7 % | **39.5%** | 19,761 | 6.0 % | 6,443 | 2.0 % | **47,5 %** |
|  | **60-64** | 10,435 | | 3.3 % | | 121,906 | | 38.5 % | **41.8%** | 23,217 | 7.3 % | 6,426 | 2.0 % | **51,2 %** |
|  | **65-69** | 11,304 | | 3.6 % | | 130,627 | | 41.4 % | **45.0%** | 27,736 | 8.8 % | 7,026 | 2.2 % | **56,0 %** |
|  | **70-74** | 8,114 | | 3.1 % | | 105,291 | | 39.7 % | **42.7%** | 26,664 | 10.0 % | 5,665 | 2.1 % | **54,9 %** |
|  | **75-79** | 3,760 | | 2.2 % | | 60,684 | | 36.2 % | **38.5%** | 18,190 | 10.9 % | 3,430 | 2.0 % | **51,4 %** |
|  | **80-84** | 1,324 | | 1.5 % | | 28,164 | | 32.6 % | **34.2%** | 8,199 | 9.5 % | 1,814 | 2.1 % | **45,8 %** |
|  | **85+** | 460 | | 0.7 % | | 14,907 | | 23.7 % | **24.4%** | 3,980 | 6.3 % | 1,283 | 2.0 % | **32,8 %** |
|  | **Total** | 42,639 | | 2.3% | | 691,754 | | 36.6% | **38.8%** | 146,973 | 7.8% | 38,725 | 2.0% | **48.6%** |
| **Women** | **30-34** | - | | - | | - | | - | **-** | 8,144 | 2.3 % | 768 | 0.2 % | **2,5 %** |
|  | **35-49** | - | | - | | - | | - | **-** | 10,150 | 2.8 % | 1,215 | 0.3 % | **3,1 %** |
|  | **40-44** | - | | - | | - | | - | **-** | 17,164 | 3.8 % | 2,744 | 0.6 % | **4,4 %** |
|  | **45-49** | - | | - | | - | | - | **-** | 19,588 | 4.8 % | 3,436 | 0.8 % | **5,6 %** |
|  | **50-54** | 875 | | 0.3 % | | 125,850 | | 37.4 % | **37.7%** | 16,240 | 4.8 % | 6,682 | 2.0 % | **44,5 %** |
|  | **55-59** | 4,898 | | 1.5 % | | 149,303 | | 45.9 % | **47.4%** | 16,291 | 5.0 % | 6,624 | 2.0 % | **54,4 %** |
|  | **60-64** | 9,767 | | 2.9 % | | 154,227 | | 46.0 % | **48.9%** | 19,547 | 5.8 % | 7,084 | 2.1 % | **56,9 %** |
|  | **65-69** | 10,942 | | 3.0 % | | 172,830 | | 47.6 % | **50.6%** | 24,192 | 6.7 % | 7,927 | 2.2 % | **59,4 %** |
|  | **70-74** | 8,425 | | 2.5 % | | 149,472 | | 44.3 % | **46.8%** | 24,899 | 7.4 % | 6,873 | 2.0 % | **56,2 %** |
|  | **75-79** | 4,129 | | 1.7 % | | 94,383 | | 39.2 % | **40.9%** | 18,138 | 7.5 % | 4,841 | 2.0 % | **50,4 %** |
|  | **80-84** | 1,394 | | 0.9 % | | 47,017 | | 31.7 % | **32.7%** | 8,940 | 6.0 % | 2,941 | 2.0 % | **40,7 %** |
|  | **85+** | 478 | | 0.3 % | | 29,395 | | 20.4 % | **20.8%** | 4,938 | 3.4 % | 2,568 | 1.8 % | **26,0 %** |
|  | **Total** | 40,908 | | 1.8% | | 922,477 | | 41.4% | **43.2%** | 133,185 | 6.0% | 45,540 | 2.0% | **51.2%** |
| **TOTAL** |  | 83,547 | | 2.0% | | 1,614,231 | | 39.2% | **41.2%** | 280,158 | 6.8% | 84,265 | 2.0% | **50.0%** |

sFOBT – screening FOBT, sC – screening colonoscopy, dFOBT – diagnostic FOBT, dC – diagnostic colonoscopy;

a contribution to complete coverage by examinations
